# Z-Ligustilide Exerted Hormetic Effect on Growth and Detoxification Enzymes of* Spodoptera litura* Larvae

**DOI:** 10.1155/2018/7104513

**Published:** 2018-07-02

**Authors:** Yang Yi, Guojun Dou, Zanyang Yu, Hui He, Chengqiang Wang, Li Li, Jia Zhou, Dejun Liu, Jianyou Shi, Guanrong Li, Lei Pang, Na Yang, Qinwan Huang, Hongyi Qi

**Affiliations:** ^1^College of Pharmaceutical Sciences, Southwest University, Chongqing 400716, China; ^2^Sichuan Institute of Food and Drug Control, Chengdu 611731, Sichuan, China; ^3^College of Chinese Medicine, Chengdu University of Traditional Chinese Medicine, Chengdu 610075, Sichuan, China; ^4^Pharmacy Department, Sichuan Academy of Medical Sciences and Sichuan Provincial People's Hospital, Chengdu 610212, Sichuan, China; ^5^College of Agronomy and Biotechnology, Southwest University, Chongqing 400716, China; ^6^Institute of Laboratory Animal Sciences, Sichuan Academy of Medical Sciences and Sichuan Provincial People's Hospital, Chengdu 610212, Sichuan, China

## Abstract

Plants have evolved a variety of phytochemicals to defense insect feeding, whereas insects have also evolved diverse detoxification enzymes, which are adaptively induced as a prosurvival mechanism. Herein, Z-ligustilide in* Ligusticum chuanxiong* Hort. was found to exhibit a similar trend in the accumulation from December to May as the occurrence of* Spodoptera litura* (Fabricius) larvae. Importantly,* S. litura *larvae feeding enhanced Z-ligustilide level in the stem and leaf (*p* < 0.01). Moreover, Z-ligustilide ranging from 1 to 5 mg·g^−1^ exhibited remarkable larvicidal activity, antifeedant activity, and growth inhibition against* S. litura *larvae. The LC_50_ values of larvicidal activity for phthalides in* L. chuanxiong* were compared as follows: Z-ligustilide > levistilide A > senkyunolide A > 3-butylidenephthalide > senkyunolide I, implicating the critical role of conjugated structure. Notably, there was a biphasic dose response for glutathione S-transferase (GST), cytochrome P450 (CYP) 450, Acetylcholinesterase (AChE), and Carboxylesterase (CarE) activities and GSTs1, cytochrome P450 (CYP) 4S9, and CYP4M14 mRNA expression. Particularly, low dose (0.1 mg·g^−1^) of Z-ligustilide conferred the resistance of* S. litura *larvae against chlorpyrifos (*p* < 0.05). Together, our data suggest that Z-ligustilide may function in a hormetic way in the chemical defense of* L. chuanxiong* against* S. litura *larvae.

## 1. Background

Plants have evolved a variety of phytochemicals that play a chemical defense role against diverse stresses, particularly insect feeding [1, 2]. It has been well-established that noxious phytochemicals may serve as botanical insecticides due to their good efficacy and environmentally friendly [2]. Importantly, it has also been gradually recognized that phytochemicals produced by plants under stress environment can activate mild cellular stress response mechanisms at a subtoxic level in humans, which may enhance the tolerance against severe dysfunction or disease [3–5], which belongs to a phenomenon of hormesis that is characterized by a biphasic dose response [6]. This is supported by the fact that humans share a great deal of biological similarities to insects, which has established a close coevolutionary relationship with plants over 4 billion years, due to the common ancestor of all multicellular organisms, evolutionary conservation, and adaptive convergence [7, 8].

*Ligusticum chuanxiong* Hort. is a well-known Chinese medicinal and edible plant of the family Apiaceae and mainly produced in Sichuan province [9].* Rhizoma Chuanxiong* is the dried rootstock of* L. chuanxiong* and traditionally used as an herbal medicine with beneficial effects for stroke, migraine, and gynecological disorders. Moreover,* Rhizoma Chuanxiong *is frequently stewed with chicken or fish as food therapy. Z-Ligustilide (Z-LIG) is a phthalide compound and accounts for the highest content in the essential oil of* L. chuanxiong* [10]. Recently, we and other investigators found that Z-LIG is one of main components responsible for the neuroprotection of* Rhizoma Chuanxiong* against ischemic stroke [11–16]. In particular, our investigations demonstrated that pretreatment with Z-LIG remarkably enhanced the tolerance of neuron-like PC12 cells and rats against ischemic injury through activating the cellular stress response pathways Nrf2 and HSP70 [14–16]. Thus, it is possible that Z-LIG belongs to hormetic phytochemicals. In previous screening studies of agrochemicals, Z-LIG exhibited strong insecticidal activity against* Drosophila melanogaster *[17, 18], mosquito larvae [19], and* Bemisia tabaci *[20]. However, it is still totally unknown whether Z-LIG plays a defense role in* L. chuanxiong* against diverse stresses, especially, insect feeding.

*Spodoptera litura* (Fabricius) (Lepidoptera: Noctuidae) is a regular polyphagous and damaging insect and distributed in the tropical and subtropical areas around the world [21, 22]. The wide outbreak of* S. litura *may be attributed to its migration, high reproductive performance, and detoxification mechanisms [23, 24]. Most recently, a recombinant protein of *α*-amylase/subtilisin inhibitor cloned from* Rhizoma Chuanxiong *exhibited potent inhibition against *α*-amylase and subtilisin A from* S. litura *larvae, indicating the defense mechanisms against* S. litura* existing in* L. chuanxiong* [22]. Curiously, little attention until now has been given to the occurrence of* S. litura *larvae in* L. chuanxiong *and the chemical defense role of phytochemicals, such as Z-LIG, in* L. chuanxiong* against* S. litura *larvae.

In the current study, we first determined the potential relationship between the accumulation of Z-LIG and the occurrence of* S. litura *larvae in* L. chuanxiong*. Then, the larvicidal activity, antifeedant activity, and growth inhibition of Z-LIG against* S. litura *larvae were evaluated. Moreover, influence of Z-LIG on the activities and/or the expression of detoxification enzymes in* S. litura *larvae was further examined. Finally, whether low dose of Z-LIG leads to the resistance of* S. litura *larvae against chlorpyrifos was particularly explored.

## 2. Materials and Methods

### 2.1. Chemicals

Z-LIG, levistilide A, senkyunolide A, senkyunolide I, and 3-butylidenephthalide were obtained from Chengdu Chroma-Biotechnology Co., Ltd. (Chengdu, China), and stored at -80°C before use. Chlorpyrifos (O,O-diethyl O-3,5,6-trichloro-2-pyridyl phosphorothioate, CPF) was purchased from J&K Scientific Ltd. (Beijing, China).

### 2.2. Extraction of Essential Oil

The plant material of* L. chuanxiong* was collected and separated as two parts, the rhizome and the stem and leaf. After weighing, the samples were extracted through the hydrodistillation process in a Clevenger Apparatus with continuous ice-cold water circulation in condenser. The volatile fraction was collected in dichloromethane and concentrated by a Buchi rotavapour system with water bath temperature no more than 25±1°C.

### 2.3. Gas Chromatography-Mass Spectrometry (GC-MS)

The Z-LIG level in in the essential oils was analyzed by GC-MS. The system consists of an Agilent 6890 type gas chromatograph and an Agilent 5975N mass spectrometer, which equips with an Agilent HP-5MS 5% Phenyl Methyl Siloxane (30 m × 0.25 *μ*m × 250 *μ*m). The analytical conditions are shown as follows: oven and initial temperature = 60°C for 3 min, ramp at 8°C/min to 180°C, then ramp at 5°C/min to 240°C; injection temperature = 230°C; injection volume = 1 *μ*L (1:1000 diluted in acetone); carrier gas = He; flow rate = 1.5 mL/min; solvent delay= 0 min; transfer temperature = 250°C; source temperature = 230°C; ionization, electron impact; ion energy = 70 eV; scan range, 30-500 Da. To quantify the content of Z-LIG, standard compound of Z-LIG with purity more than 98% was used in GC-MS analysis. Z-LIG was identified based on the retention time and mass spectral comparison with standard compound.

### 2.4. Insect Rearing

Eggs of* S. litura *were originally collected from field of* L. chuanxiong*. Then, the rearing was carried out in our laboratory under constant conditions: 27 ± 1°C, RH 60-70%, and 12L:12D photoperiod. To maintain the generation of insects, female and male moths with the same number were caged in a 10 cm diameter beaker supplied with honey solution (10%) and permitted to copulate for 48 h. Eggs were then laid by female moths on wax paper. Upon egg hatching,* S. litura* larvae were fed on standard artificial diet [25].

### 2.5. Larvicidal Activity

Test compounds were dissolved in 200 *μ*L of DMSO and mixed thoroughly with 100g artificial diet. The artificial diet with DMSO only was used as control diet. The artificial diet with different doses of compounds or control diet was placed into Petri dishes (9 cm × 1.5 cm), respectively. The third instar larvae that already starved for 4 h were introduced into each Petri dish. After 72 h, the diet was replaced with normal diet. Another 6 days later, mortality of the larvae was checked, and larvae showed that the inability to move was scored as dead. A total of 20 larvae were used in each triplicate. Abbott's formula [26] was applied to calculate the corrected mortality as follows:* P*=(*T*-*C*)/(100-*C*)×100, where* P* refers to % corrected mortality,* T* refers to % death in treatment, and* C* refers to % death in vehicle control.

### 2.6. Antifeedant Activity

Both selective antifeedant activity and nonselective antifeedant activity were evaluated in our study. For the determination of selective antifeedant activity, the test compound-containing artificial diet and the control diet were simultaneously placed into each Petri dish with 3 cm distance. Twenty of the third instar larvae that already starved for 4 h were introduced into each Petri dish. The number of larvae within 1 cm range of the diet was counted within 2 to 72 h after the introduction of larvae. The selective antifeedant rate (%) is calculated as (*C*-*T*)/(*C*+*T*) ×100, where* C* refers to the average larvae number of the control group and* T* refers to the average larvae number of the treated group. For the determination of nonselective antifeedant activity, one piece of the test compound-containing artificial diet or the control diet was placed into a Petri dish. Ten of the third instar larvae that already starved for 4 h were introduced into each Petri dish. After 48 h, the nonselective antifeedant rate (%) is calculated as (*C*-*T)/C* ×100, where* C *refers to the average diet weight of the control group before and after the bioassay and* T* refers to the average diet weight of the treated group before and after the bioassay.

### 2.7. Growth Inhibition

One piece of the test compound-containing artificial diet or the control diet was placed into a 5 cm diameter beaker. Ten of the third instar larvae were introduced into each beaker. After 48 h, the diet was replaced with normal diet. The larvae were continuously reared to become pupae. The average weight of pupae was then calculated.

### 2.8. Determination of Glutathione-S-Transferase (GST), Cytochrome P450 (CYP) CYP450, Acetylcholinesterase (AChE), and Carboxylesterase (CarE) Activities

The fifth instar larvae were exposed to the test compound-containing artificial diet or the control diet for 48 h. Midguts separated from larvae of each treatment were homogenized in liquid nitrogen and then added with 5 volumes of 0.1M phosphate buffer (pH 7.0). Each sample was centrifugated at 8000g for 10 min at 4°C. Then, the activities of GST, AChE, CYP450, and CarE were further determined with the respective commercial kits (Nanjing Jiancheng Biotechnology, China) following the instructions provided by manufacturer.

### 2.9. Semiquantitative RT-PCR Detection of GSTs1, Cytochrome P450 (CYP) 4M14, and CYP4S9

The fifth instar larvae were exposed to the test compound-containing artificial diet or the control diet for 48 h. Total RNA was isolated from midguts of* S. litura* larvae by using TRIzol® reagent (Invitrogen) and liquid nitrogen. The RT-PCR was performed with PrimeScript™ RT reagent Kit and Premix Taq (Takara Bio USA, Inc., USA) following the instructions provided by the manufacturer. The primer was designed according to the sequence of each gene in GeneBank and the specific primer sequences for* GSTs1 *(HQ667936.1),* CYP4M14* (DQ352137),* CYP4S9* (DQ355383), and* Elongation factor-1α*, (*EF-1α*) (U20129) were shown in Table 1[27, 28]. EF-1*α* was used as internal control. PCR amplification was set as follows: after an initial denaturation at 94°C for 3 min, 32 cycles of 94°C for 30 s, 55.9°C for 45 s, and 72°C for 60 s. The reaction ended with a final extension step at 72°C for 10 min. PCR products were resolved by gel electrophoresis in 1.2% agarose containing Tanon® Nucleic acid dye and visualized under UV light.

### 2.10. Statistical Analysis

The data in this study were reported as means ± SD for triplicate. The LC_50_ values were evaluated using Probit analysis. A t-test or one-way ANOVA was applied to evaluate for significant differences between two groups. A* p*-value of less than 0.05 was considered to be statistically significant.

## 3. Results

### 3.1. Influence of S. litura Larvae Feeding on the Accumulation of Z-LIG in L. chuanxiong

**Figure 1:**
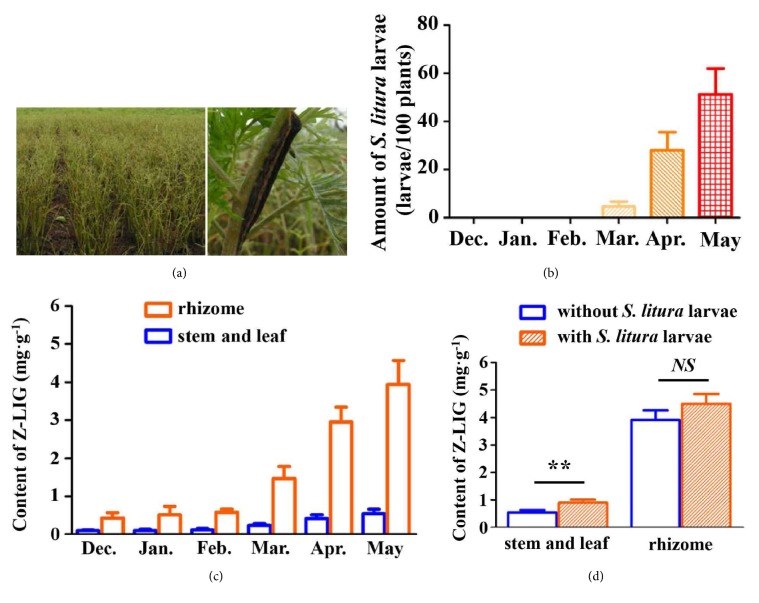
*S. litura *larvae feeding contributes to the accumulation of Z-LIG in* L. chuanxiong*. (a) Representative images showing the occurrence of* S. litura *larvae in* L. chuanxiong* in the region of Pengzhou in Sichuan province, China. (b) The incidence rate of* S. litura *larvae in* L. chuanxiong* field from December to May. (c) The accumulation of Z-LIG in* L. chuanxiong* during different growth periods. The Z-LIG content in the rhizome and the stem and leaf was analyzed by GC-MS. (d) Influence of* S. litura *larvae feeding on the content of Z-LIG in the stem and leaf and the rhizome of the potted* L. chuanxiong*. After starvation for 4 h, Third instar larvae of* S. litura* were released to feed on the potted* L. chuanxiong* (2 larvae per plants). After 24 h, the plant materials were collected and the Z-LIG content in the rhizome and the stem and leaf was analyzed by GC-MS. Values are presented as means ± SD. (*∗∗*)* p*<0.01. NS, nonsignificant.

In field observation of* L. chuanxiong* in the region of Pengzhou in Sichuan province,* S. litura* larvae were found to be one of the main pests (Figure 1(a)). To determine the incidence of* S. litura* larvae, we evaluated the amount of* S. litura* larvae in* L. chuanxiong* field during the different growth periods. As shown in Figure 1(b), no* S. litura* larvae were found during December to February. Only a few* S. litura* larvae (5 per 100 plants in average) were observed in March. However, the amount of* S. litura* larvae dramatically increased in part of* L. chuanxiong* field during April (28 larvae per 100 plants in average) and May (51 larvae per 100 plants in average). Moreover,* S. litura* larvae were found in almost 62% of* L. chuanxiong* field. Meanwhile, we also determined the accumulation of Z-LIG in* L. chuanxiong* by collecting* L. chuanxiong* from December to May and detecting the content of Z-LIG in the rhizome and the stem and leaf with GC-MS. As shown in Figure 1(c), the level of Z-LIG exhibited little change in rhizome from December to February. Obvious increase of Z-LIG in the rhizome was shown in March. A rapid accumulation of Z-LIG in the rhizome was observed in April and May, with 6.77-fold and 9.03-fold increase compared with that of December, respectively. The variation trend of Z-LIG content in the stem and leaf is very similar to that in the rhizome. However, the level of Z-LIG in the rhizome is marked higher than that in the stem and leaf, with 6.98-fold and 6.36-fold higher in April and May, respectively. Then, we determined the influence of* S. litura *larvae feeding on the content of Z-LIG in the potted* L. chuanxiong*. As shown in Figure 1(d), the Z-LIG level in the stem and leaf of the potted* L. chuanxiong*. with* S. litura *larvae feeding (2 larvae per plants for 24 h) remarkably increased compared with that without* S. litura *larvae feeding (*p *< 0.01), while the Z-LIG level in the rhizome of the potted* L. chuanxiong* with* S. litura *larvae feeding exhibited an increasing trend, but it is not statistically significant (*p > *0.05).

### 3.2. Larvicidal Activity, Antifeedant Activity, and Growth Inhibition of Z-LIG

**Figure 2:**
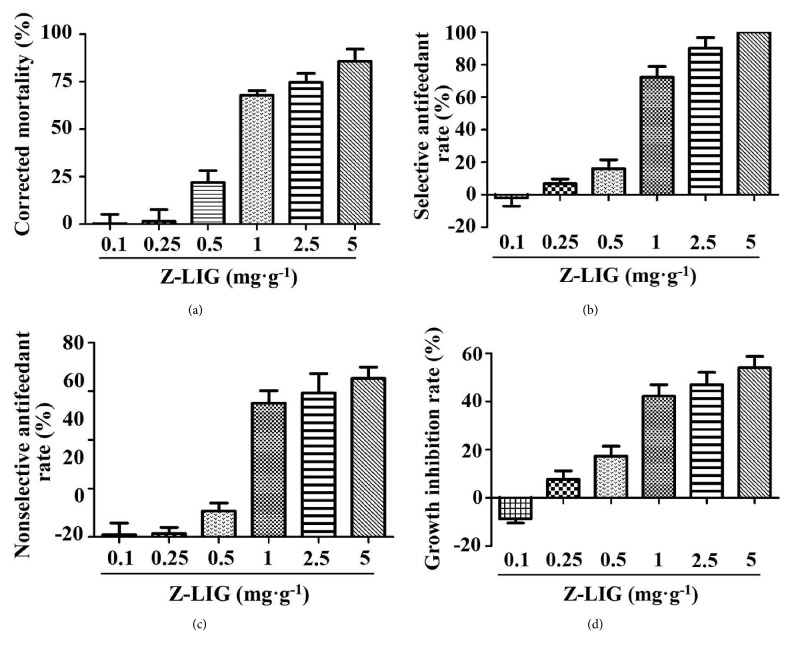
Defense effect of Z-LIG against* S. litura *larvae: (a) larvicidal activity; (b) nonselective antifeedant rate; (c) selective antifeedant rate; and (d) growth inhibition. Values are presented as means ± SD.

To determine the larvicidal activity of Z-LIG on* S. litura *larvae, we fed* S. litura *larvae with food containing different doses of Z-LIG (0.1 to 5 mg·g^−1^). As shown in Figure 2(a), 0.1 and 0.25 mg·g^−1^ of Z-LIG showed almost no insecticidal activity compared with vehicle. Moreover, 0.5 mg·g^−1^ of Z-LIG showed only weak larvicidal activity. However, Z-LIG (1 to 5 mg·g^−1^) exhibited prominent larvicidal activity. Then, we determined the antifeedant activity of Z-LIG on* S. litura *larvae. The result of selective antifeedant activity in Figure 2(b) showed that* S. litura *larvae exhibited even higher selectivity to food with 0.1 mg·g^−1^ Z-LIG than that with vehicle. Very weak selective antifeedant activity was observed for 0.25 mg·g^−1^ and 0.5 mg·g^−1^ of Z-LIG, whereas the selective antifeedant rate dramatically increased in* S. litura *larvae exposed to 1 to 5 mg·g^−1^ of Z-LIG. Moreover,* S. litura *larvae exposed to 5 mg·g^−1^ of Z-LIG exhibited 100% of selective antifeedant rate.  Figure 2(c) showed that only weak nonselective antifeedant rate was shown in* S. litura *larvae exposed to 0.1 to 0.5 mg·g^−1^ of Z-LIG. However, marked selective antifeedant rate was observed in* S. litura *larvae exposed to 1 to 5 mg·g^−1^ of Z-LIG. To further examine the effect of Z-LIG on the growth of* S. litura *larvae, we compared the weight of* S. litura *larvae fed with and without Z-LIG.  Figure 2(d) showed that 1 to 5 mg·g^−1^ of Z-LIG remarkably inhibited the weight of* S. litura *larvae and 0.25 to 0.5 mg·g^−1^ of Z-LIG only exhibited the weak or moderate inhibition, whereas 0.1 mg·g^−1^ of Z-LIG obviously promoted the growth of* S. litura *larvae.

### 3.3. Larvicidal Activity of Phthalides Existing in the Essential Oil of L. chuanxiong

**Figure 3:**
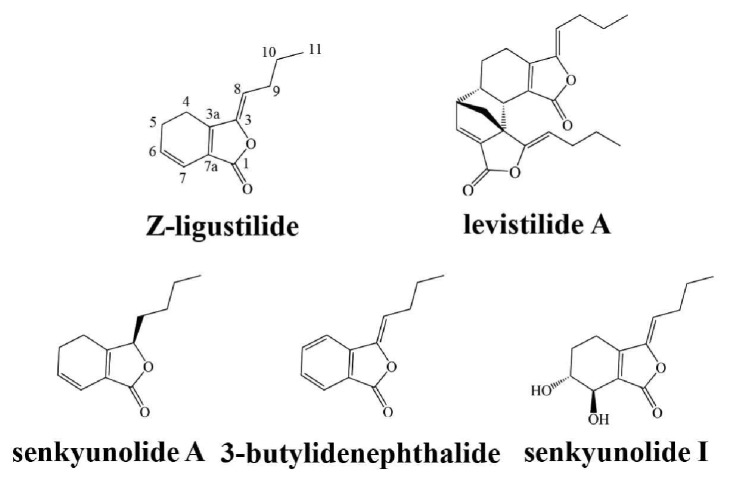
Structure of main phthalides existing in* L. chuanxiong*.

The larvicidal activity of the five phthalides (Figure 3) existing in the essential oil of* L. chuanxiong *is shown in Table 2. Z-LIG showed the most larvicidal activity among these phthalides against* S. litura *larvae, with an LC_50_ value of 0.59 mg·g^−1^. Levistilide A and senkyunolide A showed the moderate larvicidal activity, with LC_50_ values of 0.67 and 0.98 mg·g^−1^, respectively. However, both 3-butylidenephthalide and senkyunolide I were less active and exhibited LC_50_ values of 1.56 and 2.39 mg·g^−1^, respectively.

### 3.4. Effect of Z-LIG on GST, CYP450, AChE, and CarE Activities

**Figure 4:**
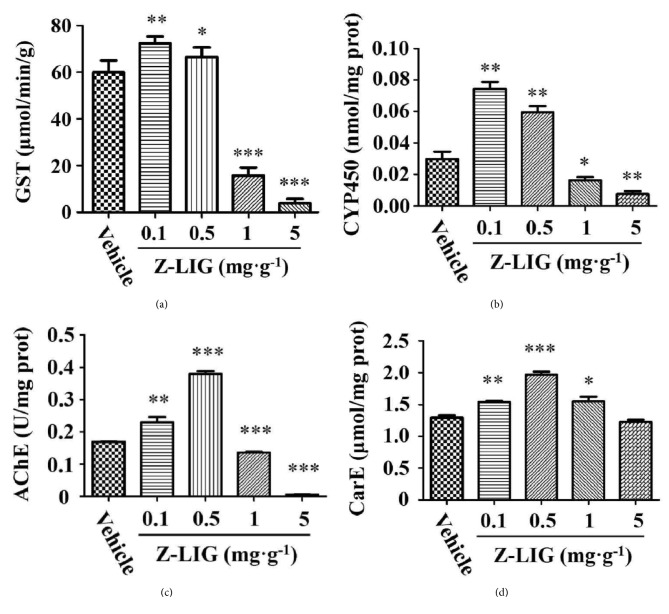
Effect of Z-LIG on the activities of detoxification enzymes GST, AChE, CYP450, and CarE. Enzyme activities were determined as mentioned under Materials and Methods. Values are presented as means ± SD. (*∗*)* p*<0.05, (*∗∗*)* p*<0.01, and (*∗∗∗*)* p*<0.001.

As shown in Figure 4(a), there was a significant increase of GST activity in* S. litura *larvae exposed to 0.1 (*p* < 0.01) and 0.5 (*p* < 0.05) mg·g^−1^ of Z-LIG compared with that exposed to vehicle, whereas GST activity dramatically decreased in* S. litura *larvae exposed to 1 and 5 mg·g^−1^ of Z-LIG (*p* < 0.001). Almost 21-fold decrease was observed in* S. litura *larvae exposed to 5 mg·g^−1^ of Z-LIG compared with that exposed to vehicle. In Figures 4(b) and 4(c), CYP450 and AChE activities also exhibited a similar trend that the activities of both enzymes remarkably increased for 0.1 and 0.5 mg·g^−1^ of Z-LIG and significantly decreased for 1 and 5 mg·g^−1^ of Z-LIG. For CarE activity, a marked increase was observed in* S. litura *larvae exposed to 0.1 to 1 mg·g^−1^ of Z-LIG compared with that exposed to vehicle and the highest enzyme activity was shown for 0.5 mg·g^−1^ of Z-LIG.

### 3.5. Effect of Z-LIG on GSTs1, CYP4S9, and CYP4M14 Expression

**Figure 5:**
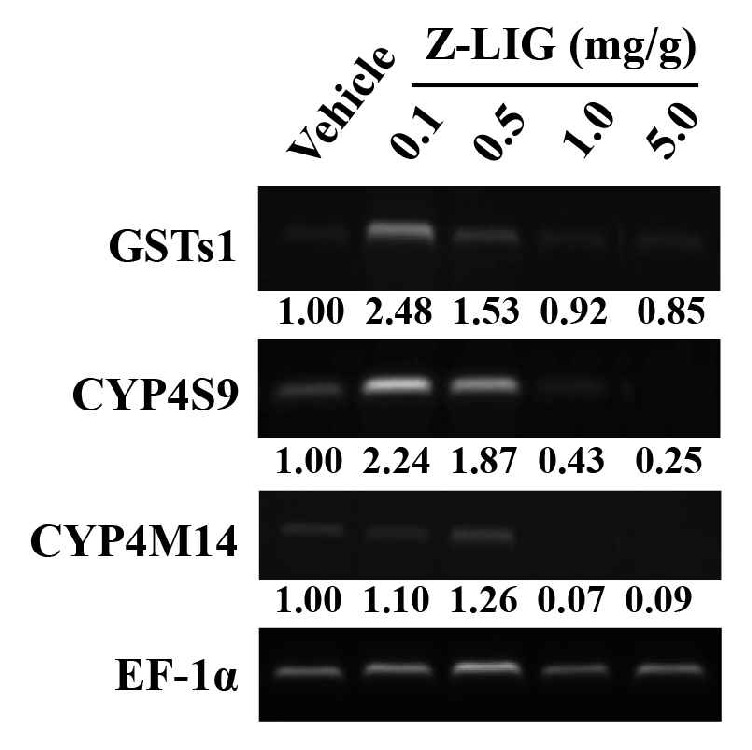
Effect of Z-LIG on the mRNA expression of* GSTS1*,* CYP4S9*, and* CYP4M14*. mRNA expression was determined as mentioned under Materials and Methods.

To further determine the influence of Z-LIG on the expression of detoxification enzymes, we used RT-PCR to evaluate the mRNA level of* GSTs1*,* CYP4S9*, and* CYP4M14*. Interestingly, the mRNA expression of all these three genes exhibited typic biphasic dose responses after* S. litura *larvae were exposed to Z-LIG (0.1 to 5 mg·g^−1^) or vehicle (Figure 5). The maximum expression for both* GSTs1 *and* CYP4S9* was observed in* S. litura *larvae exposed to 0.1 mg·g^−1^ of Z-LIG, while 0.5 mg·g^−1^ of Z-LIG caused maximum expression of* CYP4M14*.

### 3.6. Low Dose of Z-LIG Enhanced Resistance of S. litura Larvae against Pesticide CPF

**Figure 6:**
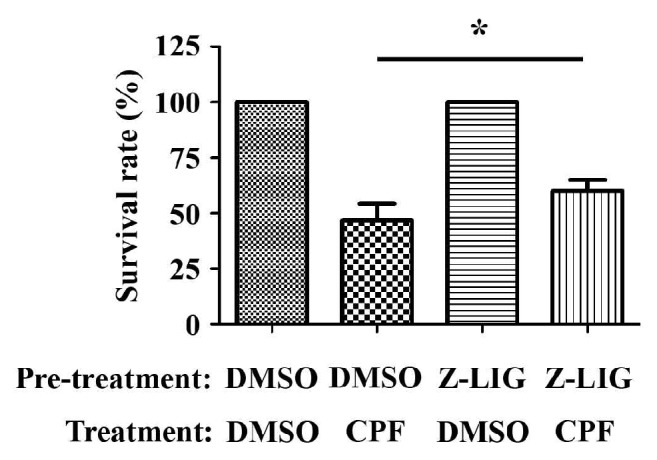
Low dose of Z-LIG confers resistance of* S. litura *larvae against CPF.* S. litura *larvae were pretreated with 0.1 mg·g^−1^ of Z-LIG or vehicle for 48 h and then treated with CPF (2.5 *μ*g·g^−1^) or vehicle for another 3 days and the survival rate was determined. Values are presented as means ± SD; (*∗*)* p*<0.05.

To determine the possible influence of low dose of Z-LIG on the resistance of* S. litura *larvae, we pretreated* S. litura *larvae with 0.1 mg·g^−1^ of Z-LIG or vehicle for 48 h and then treated* S. litura *larvae with CPF (2.5 *μ*g·g^−1^) or vehicle for another 3 days and the survival rate was determined. As shown in Figure 6, there was no* S. litura *larvae death in the group pretreated and treated with vehicle. The same result was also observed in the group pretreated with Z-LIG and treated with vehicle. However, there was only 46.67% survival rate in the group of vehicle pretreatment and CPF treatment. Notably, the survival rate significantly increased to 60 % in the group of Z-LIG pretreatment and CPF treatment (*p* < 0.05). These results suggest that low dose of Z-LIG pretreatment may enhance the resistance of* S. litura *larvae against CPF.

## 4. Discussion

In an ecological environment, plants have evolved sophisticated defense systems to sense and resist a variety of biotic threats. Invertebrates, particularly insects, are the major biotic factor that interacts with plants in the microenvironment as insects have evolved to feed on plants at least 4 billion years ago, which leads to the formation of a close coevolutionary relationship [29]. The main approach employed by plants to avoid the attack from herbivorous insects is the chemical defense, which is partially mediated by the production of toxic and repellent compounds that accumulate after insect feeding [30, 31]. Recently, we directed our effort to screen hormetic phytochemicals from essential oils extracted from traditional Chinese herbal medicine. As a result, a group of phthalide compounds isolated from the essential oil of* Rhizoma Chuanxiong* drew our special attention. Among them, Z-LIG exhibited potent neuroprotective effect against ischemic injury via the activation of cellular stress response mechanism Nrf2 and HSP70 pathways [14–16]. Meanwhile, the role that Z-LIG plays in* L. chuanxiong* is also very interesting to us.* Rhizoma Chuanxiong *collected from the regions of Dujiangyan and Pengzhou in Sichuan province is of high quality and large quantities and supplies nationwide and abroad [9]. It has been found that* S. litura *larvae are one of the major pests that feed on* L. chuanxiong* distributed in Dujiangyan and Pengzhou [32]. Thus, the current study aimed to figure out whether Z-LIG plays a defense role in* L. chuanxiong* against* S. litura *larvae.

In the field of* L. chuanxiong* in Pengzhou of Sichuan province, we found that* S. litura *larvae were indeed one of the main pests of* L. chuanxiong*. Thus, we first determined the incidence of* S. litura* larvae in* L. chuanxiong* and the accumulation of Z-LIG in the rhizome and the stem and leaf during the different periods of growth. Interestingly, we observed that there was a quite similar increasing trend for Z-LIG content and* S. litura* larvae amount, both of which showed a rapid growth during March to May. Then, we asked whether feeding by* S. litura *larvae influences the accumulation of Z-LIG in* L. chuanxiong*. Interestingly, our results showed that* S. litura* larvae feeding led to the increase of Z-LIG in the stem and leaf of the potted* L. chuanxiong*, suggesting that Z-LIG accumulation in* L. chuanxiong* may be at least in part correlated with the outbreak of* S. litura *larvae. Similarly, accumulating evidence have demonstrated that phytochemicals are adaptively induced and accumulate in plants in response to attack by insects. For example, the induced accumulation of isoflavone 7-O-glucosides and isoflavone 7-O-(6′′-O-malonyl-*β*-glucosides) was observed after soybean (Var. Enrei) leaves were treated with a combination of plant defense elicitors present in* S. litura* gut content extracts [33]. Two isoflavone aglycones (daidzein and formononetin) and their conjugates (glucosides and malonyl glucosides) were found to increase in soybean leaves [Glycine max (L.) Merr. (Fabaceae)] following attack by common cutworm larvae [34]. The highly toxic isothiocyanates were produced by the myrosinase mediated hydrolysis of glucosinolates in cruciferous plants upon the damage of herbivores [35]. We then evaluated whether Z-LIG showed a defense effect against* S. litura *larvae by determining larvicidal activity, antifeedant activity, and growth inhibition. Our results showed that Z-LIG ranging from 0.1 to 0.5 mg·g^−1^ showed no or weak larvicidal activity, antifeedant activity, and growth inhibitory activity against* S. litura *larvae, whereas higher dose (1 to 5 mg·g^−1^) of Z-LIG exhibited remarkable larvicidal activity, antifeedant activity, and growth inhibitory activity. Previous studies demonstrated that Z-LIG also exhibited strong insecticidal activity against other insects. For example, Z-LIG was shown to be the most potent toxicity among the alkylphthalides isolated from the chloroform extract of* Cnidium officinale* rhizomes against the larvae and adults of Drosophila melanogaster [17, 18] and the Q-biotype females of* Bemisia tabaci *[20]. Besides Z-LIG, we also determined the larvicidal activity of other structure-similar phthalides existing in the essential oil of* L. chuanxiong*. Based on LC_50_ values, the potency of the larvicidal activity of these phthalides against* S. litura *larvae can be summarized as follows: Z-Ligustilide > levistilide A > senkyunolide A > 3-butylidenephthalide > senkyunolide I. The structure-activity relationship can be further drawn based on the results. Z-LIG bears a typical *α*, *β*, *γ*, *δ*-unsaturated lactone. Moreover, the carbonyl group at its C-2 position is an electron-withdrawing group, which can withdraw electron from the *α*, *β*, *γ*, *δ*-unsaturated double bonds. Thus, the unsaturated double bond between C-3 and C-8 in Z-LIG is highly active. Levistilide A is polymerized by two molecular Z-LIG and only one complete chain of *α*, *β*, *γ*, *δ*-unsaturated double bonds is preserved. Senkyunolide A lacks *γ*, *δ*-unsaturated double bond compared with Z-LIG. The aromatic ring of 3-butylidenephthalide is a strong electron donating group, which greatly reduces the reactive ability of the *γ*, *δ*-unsaturated double bonds. The hydroxyl group at the C-7 position of senkyunolide I can form a conjugated effect with *α*, *β*, *γ*, *δ*-two double bonds. Under this effect, hydroxyl group is an electron donating group, which also reduces the reactive ability of the *γ*, *δ*-unsaturated double bonds. Thus, our results indicate the presence of conjugation of *α*, *β*, *γ*, *δ*-unsaturated double bonds with carbonyl group rather than aromaticity or hydroxyl group seems to play a critical role for the larvicidal activity of phthalides against* S. litura *larvae. Consistent with our results, Chae et al. [20] reported that the conjugation is highly correlated with the toxicity of three phthalides identified in* Rhizome Cnidium officinale* (Japanese* Chuanxiong*) to the Q-biotype females. However, it has also been shown that the conjugation and aromaticity seem to play an important role in the larvicidal and adulticidal activity of phthalides against* Drosophila melanogaster *[17, 18].

Notably, the effect of Z-LIG on the enzyme activities of GST, CYP450, AChE, and CarE in* S. litura *larvae showed a biphasic dose response with an increase in low dose of Z-LIG and decrease in high dose of Z-LIG, which is supported by Z-LIG-mediated change of* GSTS1 *mRNA expression, which also exhibited a biphasic dose response. Similar trend was further observed in the mRNA expression of* CYP4S9* and* CYP4M14 *in* S. litura *larvae after Z-LIG treatment. These results indicate the possibility that detoxification enzymes can be adaptively induced in* S. litura *larvae as a prosurvival mechanism in response to low dose of Z-LIG, whereas they were directly inhibited under the insult of high dose of Z-LIG, which further confirms the hormetic role of Z-LIG. Our previous studies showed that low dose of Z-LIG induced transcription factor Nrf2-mediated detoxification enzymes and HSP70 pathways, which resulted in the tolerance against ischemic injury [14–16]. We are then curious about whether low dose of Z-LIG enhances the resistance of* S. litura *larvae as we already showed that low dose of Z-LIG induced the expression and/or activity of detoxification enzymes in Figures 4 and 5. Interestingly, our results clearly demonstrated that the resistance of* S. litura *larvae against pesticide CPF was remarkably enhanced by the pretreatment of low dose (0.1 mg·g^−1^) of Z-LIG. This result obtained from the level of plant and insect interaction may provide an explanation for our recent study, in which subtoxic dose of Z-LIG activated the cellular stress response mechanisms, such as Nrf2 and HSP70 mediated pathways, in rats, and enhanced the tolerance of the rats against ischemic injury [14–16].

Under field conditions,* S. litura *larvae feed mainly on leaf and occasionally on stem of* L. chuanxiong*. In our study, we found that Z-LIG level in the stem and leaf of* L. chuanxiong* was 0.42 ± 0.10 mg·g^−1^ in April and 0.55 ± 0.11 mg·g^−1^ in May. Under our experimental conditions, Z-LIG ranging from 0.1 to 0.5 mg·g^−1^ showed weak larvicidal activity, antifeedant activity, and growth inhibitory activity against* S. litura *larvae. Meanwhile, the detoxification enzymes in the midgut of* S. litura *larvae were also adaptively triggered by Z-LIG ranging from 0.1 to 0.5 mg·g^−1^. Thus, it can be reasonably speculated that Z-LIG produced in the stem and leaf of* L. chuanxiong* may play a weak chemical defense role against* S. litura *larvae, while* S. litura *larvae also utilize detoxification enzymes as prosurvival mechanism against the toxicity of Z-LIG, which implicating an interaction between* L. chuanxiong* and* S. litura *larvae.

## 5. Conclusion

Our study demonstrated that high dose of Z-LIG exhibited remarkable larvicidal activity, antifeedant activity, and growth inhibition against* S. litura *larvae and dramatically inhibited the activity and/or expression of detoxification enzymes in* S. litura *larvae. However, low dose of Z-LIG relevant to the level of Z-LIG in* L. chuanxiong* showed a weak defense effect on* S. litura *larvae, which was accompanied by the adaptive induction of detoxification enzymes in* S. litura *larvae. These results suggest that Z-LIG may function as a hormetic phytochemical in the chemical defense of* L. chuanxiong* against* S. litura *larvae, which may facilitate our understanding of health benefits of Z-LIG at subtoxic dose in humans from ecological and evolutionary perspective.

## Figures and Tables

**Table 1:** Primers used for semiquantitative RT-PCR.

Genes	Primer sequences
*GSTs1* (HQ667936.1)	F 5′-AACATTGTCGTCATTGGACA-3′ R 5′-ACTTGCTGGTCTCAAAT TTC-3′
*CYP4M14* (DQ352137)	F5′-GTTGCTCGCTAATCATAGGAAAAT-3′ R 5′-GGTTCTTAAACAAATCTGGTCTCAA-3′
*CYP4S9* (DQ355383)	F5′-CAACGATGTCTGATCTGGCT-3′ R 5′-GCAGGTCGTATATGTGGATTGAT-3′
*EF-1α* (U20129)	F5′-ATGCCGAAATACGTATTCCACTAC-3′ R 5′-TCCATATTCTAAGCAGGAACTGCA-3′

F: forward primer; R: reverse primer.

**Table 2:** LD_50_ values for larvicidal activity of phthalides against *S. Litura *larvae.

Compound	LD_50_^*a*^ (mg·g^−1^)	95%CL^*b*^ (mg·g^−1^)	slope±SE	R^2^
Z-LIG	0.59	0.51-0.69	1.03±0.28	0.9594
senkyunolide A	0.98	0.84-1.13	0.86±0.20	0.9649
senkyunolide I	2.39	2.04-2.80	0.91±0.23	0.9611
levistilide A	0.67	0.58-0.76	0.93±0.21	0.9688
3-butylidenephthalide	1.56	1.35-1.81	0.88±1.21	0.9657

^a^LC_50_ is the lethal concentration for 50% mortality;  ^b^95% confidence limit.

## Data Availability

Access to the data used to support the findings of this study can be considered by the author upon request, with permission of institutional review board. Please contact the corresponding author for the requests.

## Abbreviations

CYP: Cytochrome P450
CPF: Chlorpyrifos
GST: Glutathione S-transferase
GC-MS: Gas chromatography-mass spectrometry
RT-PCR: Reverse transcriptase-polymerase chain reaction
Z-LIG: Z-ligustilide.

## References

[1] Harborne J. B. (2007). Role of Secondary Metabolites in Chemical Defence Mechanisms in Plants. *Ciba Foundation Symposium 154 - Bioactive Compounds from Plants*.

[2] Miresmailli S., Isman M. B. (2014). Botanical insecticides inspired by plant-herbivore chemical interactions. *Trends in Plant Science*.

[3] Son T. G., Camandola S., Mattson M. P. (2008). Hormetic dietary phytochemicals. *NeuroMolecular Medicine*.

[4] Calabrese V., Cornelius C., Dinkova-Kostova A. T. (2012). Cellular stress responses, hormetic phytochemicals and vitagenes in aging and longevity. *Biochimica et Biophysica Acta*.

[5] Murugaiyah V., Mattson M. P. (2015). Neurohormetic phytochemicals: an evolutionary-bioenergetic perspective. *Neurochemistry International*.

[6] Mattson M. P. (2008). Hormesis defined. *Ageing Research Reviews*.

[7] Kennedy D. O., Wightman E. L. (2011). Herbal extracts and phytochemicals: plant secondary metabolites and the enhancement of human brain function. *Advances in Nutrition*.

[8] Kultz D. (2005). Molecular and evolutionary basis of the cellular stress response. *Annual Review of Physiology*.

[9] Zhao Z., Guo P., Brand E. (2012). The formation of daodi medicinal materials. *Journal of Ethnopharmacology*.

[10] Yan R., Li S. L., Chung H. S., Tam Y., Lin G. (2005). Simultaneous quantification of 12 bioactive components of *Ligusticum chuanxiong* Hort. by high-performance liquid chromatography. *Journal of Pharmaceutical and Biomedical Analysis*.

[11] Kuang X., Yao Y., Du J. R., Liu Y. X., Wang C. Y., Qian Z. M. (2006). Neuroprotective role of Z-ligustilide against forebrain ischemic injury in ICR mice. *Brain Research*.

[12] Kuang X., Du J.-R., Liu Y.-X., Zhang G.-Y., Peng H.-Y. (2008). Postischemic administration of Z-Ligustilide ameliorates cognitive dysfunction and brain damage induced by permanent forebrain ischemia in rats. *Pharmacology Biochemistry & Behavior*.

[13] Wu X.-M., Qian Z.-M., Zhu L. (2011). Neuroprotective effect of ligustilide against ischaemia-reperfusion injury via up-regulation of erythropoietin and down-regulation of RTP801. *British Journal of Pharmacology*.

[14] Yu J., Jiang Z., Ning L. (2015). Protective HSP70 induction by Z-ligustilide against oxygen-glucose deprivation injury via activation of the MAPK pathway but not of HSF1. *Biological & Pharmaceutical Bulletin*.

[15] Qi H., Han Y., Rong J. (2012). Potential roles of PI3K/Akt and Nrf2-Keap1 pathways in regulating hormesis of Z-ligustilide in PC12 cells against oxygen and glucose deprivation. *Neuropharmacology*.

[16] Li J., Yu J., Ma H. (2017). Intranasal Pretreatment with Z-Ligustilide, the Main Volatile Component of Rhizoma Chuanxiong, Confers Prophylaxis against Cerebral Ischemia via Nrf2 and HSP70 Signaling Pathways. *Journal of Agricultural and Food Chemistry*.

[17] Miyazawa M., Tsukamoto T., Anzai J., Ishikawa Y. (2004). Insecticidal effect of phthalides and furanocoumarins from Angelica acutiloba against Drosophila melanogaster. *Journal of Agricultural and Food Chemistry*.

[18] Tsukamoto T., Ishikawa Y., Miyazawa M. (2005). Larvicidal and adulticidal activity of alkylphthalide derivatives from rhizome of Cnidium officinale against Drosophila melanogaster. *Journal of Agricultural and Food Chemistry*.

[19] Wedge D. E., Klun J. A., Tabanca N. (2009). Bioactivity-guided fractionation and GC/MS fingerprinting of Angelica sinensis and Angelica archangelica root components for antifungal and mosquito deterrent activity. *Journal of Agricultural and Food Chemistry*.

[20] Chae S.-H., Kim S.-I., Yeon S.-H., Lee S.-W., Ahn Y.-J. (2011). Adulticidal activity of phthalides identified in cnidium officinale rhizome to B- and Q-biotypes of bemisia tabaci. *Journal of Agricultural and Food Chemistry*.

[21] Hou Q. (2004). The correlations of the different host plants with preference level, life duration and survival rate of Spodoptera litura Fabricius. *Chinese Journal of Eco-agriculture*.

[22] Yu J., Li Y., Xiang M. (2017). Molecular cloning and characterization of *α*-amylase/subtilisin inhibitor from rhizome of Ligusticum chuanxiong. *Biotechnology Letters*.

[23] Liu J., Zheng S., Liu L., Li L., Feng Q. (2010). Protein profiles of the midgut of spodoptera litura larvae at the sixth instar feeding stage by shotgun ESI-MS approach. *Journal of Proteome Research*.

[24] Baskar K., Ignacimuthu S. (2012). Antifeedant, larvicidal and growth inhibitory effects of ononitol monohydrate isolated from *Cassia tora* L. against *Helicoverpa armigera* (Hub.) and *Spodoptera litura* (Fab.) (Lepidoptera: Noctuidae). *Chemosphere*.

[25] Chen Q. J., Li J., Pang H. (2000). A simple artificial diet for mass rearing of some noctuid species. *Entomological Knowledge*.

[26] Abbott W. S. (1987). A method of computing the effectiveness of an insecticide. *Journal of the American Mosquito Control Association*.

[27] Wang R., Sun Y., Liang X. (2012). Effects of six plant secondary metabolites on activities of detoxification enzymes in Spodoptera litura. *Shengtai Xuebao/ Acta Ecologica Sinica*.

[28] Huang Y., Xu Z., Lin X., Feng Q., Zheng S. (2011). Structure and expression of glutathione S-transferase genes from the midgut of the Common cutworm, Spodoptera litura (Noctuidae) and their response to xenobiotic compounds and bacteria. *Journal of Insect Physiology*.

[29] Qi H., Li L., Ma H. (2018). Cellular stress response mechanisms as therapeutic targets of ginsenosides. *Medicinal Research Reviews*.

[30] Howe G. A., Jander G. (2008). Plant immunity to insect herbivores. *Annual Review of Plant Biology*.

[31] Mithöfer A., Boland W. (2012). Plant defense against herbivores: Chemical aspects. *Annual Review of Plant Biology*.

[32] Zeng H., Ni G., He L. (2009). Law of Occurrence and the Damage of Main Diseases and Pests in Ligusticum chuanxiong Hort Field. *Southwest China Journal of Agricultural Sciences*.

[33] Nakata R., Kimura Y., Aoki K. (2016). Inducible De Novo Biosynthesis of Isoflavonoids in Soybean Leaves by Spodoptera litura Derived Elicitors: Tracer Techniques Aided by High Resolution LCMS. *Journal of Chemical Ecology*.

[34] Murakami S., Nakata R., Aboshi T. (2014). Insect-Induced Daidzein, Formononetin and Their Conjugates in Soybean Leaves. *Metabolites*.

[35] Textor S., Gershenzon J. (2009). Herbivore induction of the glucosinolate-myrosinase defense system: Major trends, biochemical bases and ecological significance. *Phytochemistry Reviews*.

